# Sustainable Microbial Biostimulant Production by Integrated Bioreactor Fermentation and Membrane Emulsification

**DOI:** 10.1021/acssuschemeng.6c02820

**Published:** 2026-05-05

**Authors:** Emma Piacentini, Antonio Condello, Fabio Bazzarelli, Rebecca Italia, Valeria Ventorino, Donatella Cimini

**Affiliations:** † Institute on Membrane Technology (CNR-ITM), 87036 Rende, CS, Italy; ‡ Department of Agricultural Sciences, 590550 University of Naples Federico II, 80055 Portici, Italy; § Department of Environmental, Biological and Pharmaceutical Sciences and Technologies, University of Campania L. Vanvitelli, 81100 Caserta, Italy

**Keywords:** microbial biostimulant, sustainable agriculture, membrane emulsification, bioreactor fermentation, food waste

## Abstract

Plant growth-promoting microorganisms represent a sustainable alternative to chemical inputs for improving crop yield and nutritional quality. Their commercialization is limited by the lack of effective formulation technologies and fermentations. In fact, their functionality is related to the implementation of a protective matrix required to enhance microbial survival and application efficiency. To hasten their adoption, the development of advanced formulation technologies that can be easily scaled up, operate under mild conditions, and ensure cell immobilization efficiency and survival is needed. The potential of microbial-based biostimulants can be further promoted by the implementation of sustainable and efficient fermentation processes capable of yielding high concentrations of viable microbial cells. In the present study, membrane emulsification was applied as an advanced encapsulation approach to produce alginate capsules containing *Kosakonia pseudosacchari* TL13, a promising biofertilizer bacterial strain. Citrus waste biomass was converted into *K. pseudosacchari* TL13 biomass in a bioreactor fermentation, and the microorganism and the fermentation-derived bioactive compounds were simultaneously encapsulated. Results demonstrated that (i) membrane technology can be used to redesign the conventional encapsulation process based on emulsification with reduced shear stress and high efficiency in terms of formulation quality and microorganism loading and survival, (ii) high concentrations of biomass and added-value products (i.e., exopolysaccharides) can be obtained by using low-cost and easily available food waste, and (iii) the downstream of the process can be simplified by using the whole broth as material for biostimulant production. Overall, the proposed integrated strategy permits providing a sustainable process for microbial-based biostimulant production based on process efficiency and environmental impact.

## Graphical abstract

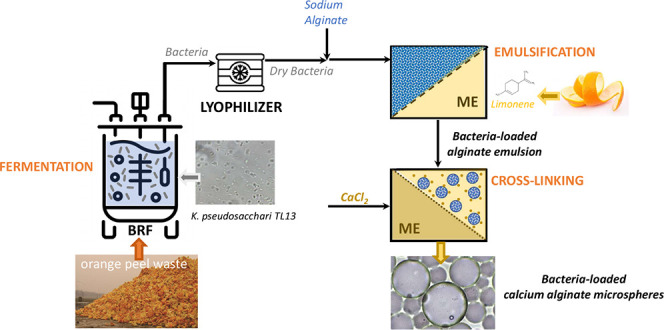

## Introduction

Microbial-based biostimulants are increasingly recognized as key components of sustainable agriculture, providing innovative strategies to improve plant growth, resilience, and productivity while reducing the environmental footprint of conventional agricultural inputs. Their large-scale deployment is accompanied by significant technical and economic challenges.

Key technological limitations included: (i) limited shelf life and product stability. Microbial-based biostimulants are sensitive to temperature, humidity, and storage conditions. Their viability declines over time, which makes long-term storage and distribution difficult without advanced formulation or packaging technologies; (ii) inconsistent quality and effectiveness. Variability in production methods and strains leads to inconsistent product quality and unpredictable field performance. Ensuring viable, high-quality microbial populations during manufacturing, storage, and application remains a technological challenge; (iii) lack of suitable carrier materials and formulations. The materials used to support and deliver microbial inoculants (carriers) are often inadequate, reducing microbial survival and efficacy. Better carrier and encapsulation technologies are needed to improve performance across diverse conditions.

In recent years, there has been increasing interest in the immobilization of plant growth-promoting microorganisms (PGPM) in various carriers to ensure their long-term survival in the soil environment. Among the carriers, polymeric materials, including alginate-based ones, have attracted considerable attention.^1^ Alginate is the most abundant polysaccharide of brown algae such as *Macrocystis pyrifera*, *Laminaria hyperborea*, *Laminaria digitata*, *Saccharina japonica*, *Lessonia nigrescens*, *Lessonia trabeculata*, *Ecklonia arborea*, *Ecklonia radiata*, *Durvillaea potatorum*, and *Ascophyllum nodosum*. It consists of various proportions of epimers of uronic acid, α-l-guluronic acid (G), and β-d-mannuronic acid (M) that are homogeneously or heterogeneously linked by 1 → 4 glycosidic bonds to form three different building blocks, polyM, polyG, and polyMG.^2^ Alginate is the most used biopolymer for microencapsulation of microbial cells for its desired properties, such as high mechanical stability, water holding capacity, nontoxicity, biodegradability, low cost, and easy availability.^3^

Emulsification is the most efficient technology for the large-scale production of encapsulated bacteria in alginate-based formulations for use in agro-industrial processes due to the absence of heat sources that can damage the bioactive agents.^4^ Membrane emulsification (ME) is a relatively new technology applied with success for the encapsulation of active molecules.^5^ Its main distinguishing aspect is that droplets are formed by a drop-by-drop mechanism without applying high disruptive forces, as in conventional emulsification devices that can compromise the structural and functional integrity of sensitive encapsulated components such as living cells.^6^ This makes the ME process highly attractive as a method for encapsulating cells, although it has still not been thoroughly investigated. The use of ME for this purpose has been reported in very few studies.^7–11^ . Probiotic bacteria such as *Lacticaseibacillus rhamnosus* GG^7^ and *Lacticaseibacillus casei* YIT 9018 ^8^ were encapsulated by ME using sodium alginate (SA) alone or with whey protein isolate, rice, and pea proteins as carrier material, respectively. The encapsulation of *Saccharomyces cerevisiae* cells in gelatin/chitosan or pure gelatin microparticles^9^ and of bacteriophages (viruses that infect and kill bacteria) in Eudragit/alginate microparticles^10,11^ using ME was also reported. Therefore, despite its high potential, ME has never been applied for the encapsulation of PGPM.

In the present study, ME is used for the production of alginate capsules containing the *Kosakonia pseudosacchari* TL13 strain, identified as a promising biofertilizer to improve agricultural production as an eco-friendly alternative to harmful chemicals.^12^ W/O emulsions obtained by using sodium alginate (SA) as dispersed phase were previously produced by ME.^7,11,13–19^ Petroleum solvents (such as kerosene,^19^ cyclohexane,^13^ isooctane,^14,18^ and paraffin oil^15,17^ ) are commonly used as the continuous phase. In the present work, petroleum solvents were replaced by a renewable solvent such as d-limonene, the major essential oil component of a byproduct from the orange juice industry.^20^ Its use as solvent extraction is well documented,^21^ demonstrating many advantages such as increased production efficiency, contribution to environmental preservation by reducing the use of solvents, fossil energy, and generation of hazardous substances. d-Limonene is also widely used as a flavoring agent and adjuvant in the food and beverage industries, as well as in cosmetics for the formulation of perfumes and other personal hygiene products.^22^ For its broad spectrum of health benefits, including anticancer, anxiolytic, anti-inflammatory, antioxidant, analgesic, antidiabetic, and antiallergic activities, it is also used as a functional ingredient in different formulations with promising applications in the agri-food industry^23,24^ elucidated to improve its possibilities to be applied as a biopesticide and preservative. Till now, its use as an oil phase in W/O emulsion has not been considered, although its hydrophobicity and biodegradability make it a valuable replacement for traditional solvents. Internal^13,15,16^ and external^7,11,14,17–19^ gelation were combined with ME to cross-link alginate emulsions by using calcium as a divalent cation. The addition of calcium solution into the emulsion determined bead coagulation into larger agglomerates due to the difficulty in controlling the gelation in a biphasic system.^25^ Internal gelation is usually used to overcome this limitation by adding an insoluble calcium salt in the dispersed phase and subsequently promoting its dissolution by lowering the pH. An alternative is to use an emulsion of CaCl_2_ in oil that, in contact with alginate droplets, causes ionic cross-linking. Only in one work was this procedure used in combination with ME.^19^

In the present work, the cross-linking of alginate emulsion by using a CaCl_2_ emulsion was studied, evaluating the effect of the CaCl_2_ emulsion composition and cross-linking reaction time.

One of the major constraints to the widespread adoption of PGPMs is their cost competitiveness relative to traditional fertilizers and pesticides. Despite their environmental benefits, biostimulants often entail high production costs correlated to their production. Due to its high organic content and large generation volume, food waste is an attractive feedstock for resource recovery. Oriented fermentation converting this waste stream into valuable products is a promising technology with high efficiency and strong application prospects to reduce food losses and pressure on global resources, and improve sustainability. After fermentation, the recovery of target products is a key problem due to the lack of green and economic methods.

In the present work, fermentation process efficiency and environmental impact were also evaluated, taking into consideration the process designed to convert citrus waste into *K. pseudosacchari* TL13 biomass and exopolysaccharide (EPS), both parts of the encapsulated material, to simplify the process and, at the same time, increase the overall sustainability of the integrated approach.

Overall, the work proposes an integrated sustainable process for microbial biostimulant production from bioreactor fermentation to encapsulation. The use of waste materials as a source for biomass production (orange peel waste) and emulsion formulation ingredients (limonene) is in line with the new environmental policies of the European Union to minimize waste and produce innovative and environmentally friendly products. It is expected that the results achieved pave the way for further investigation on PGPM encapsulation membrane-based processes as innovative technologies that ensure high crop yields and nutritional quality while mitigating the impacts of environmental change.

## Materials and Methods

### Reagents

Alginic acid sodium salt, calcium chloride, limonene, isooctane, and Span 80 were purchased from Merk Life Science S.r.l., Italy. *K. pseudosacchari* TL13 was provided by the Department of Agricultural Sciences, University of Naples Federico II, Naples, Italy.

### Alginate Emulsion Preparation by ME

The W/O emulsion of SA was prepared by using the dispersion cell (Figure 1) equipped with a circular standard disc membrane (with pores distributed across the entire surface and effective cross-sectional area of 2.76 cm^2^) with 20 μm pore size, both supplied by Micropore Technology. The hydrophobic coating on the stainless-steel membrane was obtained using octylphosphonic acid (OPA), following the protocol provided by the company. A solution of SA at 2% w/w and limonene (or Isooctane) with Span 80 2% w/w were used as dispersed and continuous phases, respectively.

**Figure 1:**
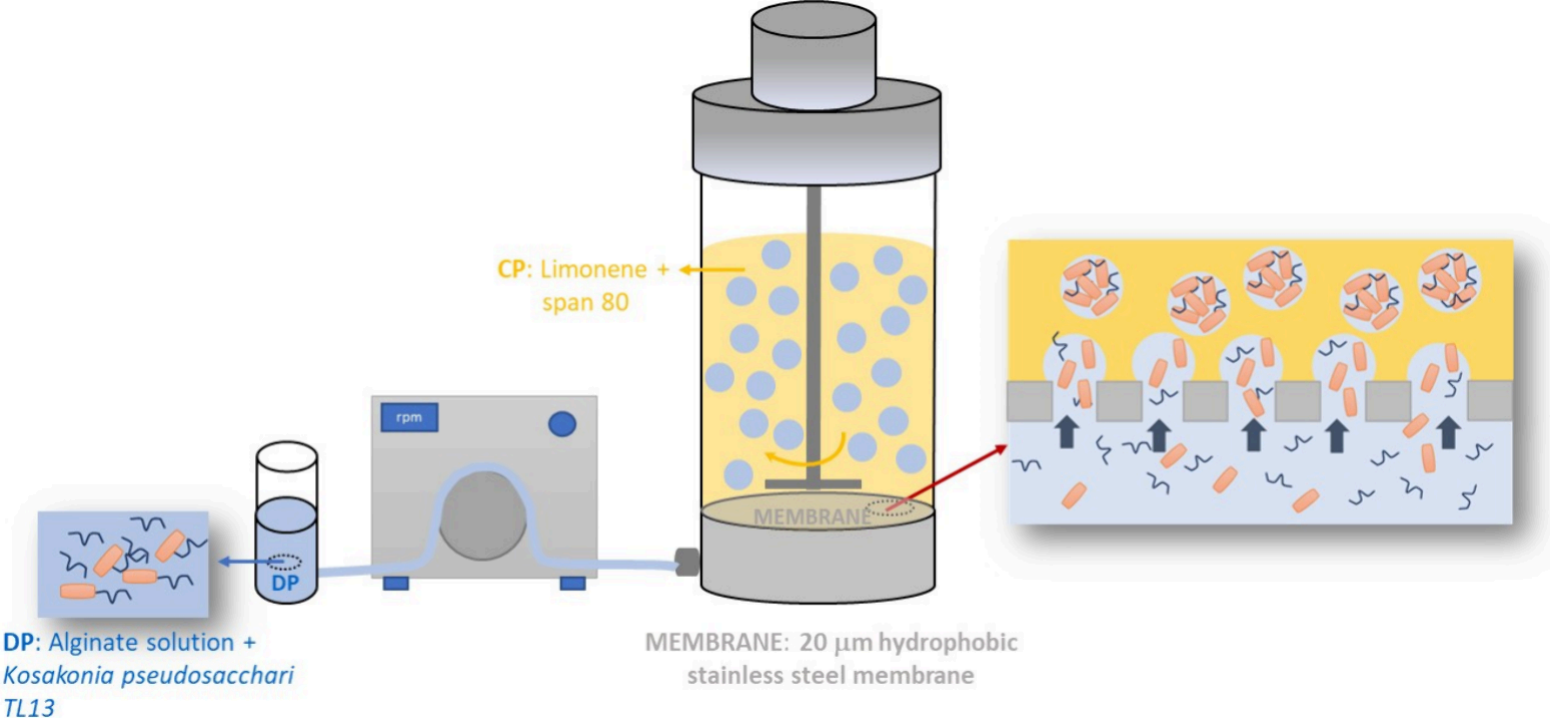
Sodium alginate emulsion production by membrane emulsification.

The effect of fluid-dynamic conditions of the emulsification process was studied. The emulsion was obtained by passing 3 mL of dispersed phase through the pores of the membrane with a flow rate in the range from 0.1 to 0.6 mL min^–1^ (which corresponds to a dispersed phase flux from 7 to 42 L h^–1^ m^–2^), while the continuous phase was agitated by setting the stirrer speed in the range from 554 to 1109 rpm (which corresponds to a shear stress of 3.5–10.7 Pa). The effect of the W/O volumetric ratio (v/v %) in the range from 10 to 30% v/v was also considered.

To encapsulate *K. pseudosacchari* TL13 in alginate emulsion droplets, 1 mL of *K. pseudosacchari* TL13 suspension in quarter-strength Ringer’s solution (VWR, Italy) containing tetrasodium pyrophosphate (16% w/v) was added to 20 mL of SA solution 2% w/w to achieve an initial amount of bacterial inoculum with respect to the amount of polymer in the range from 4 × 10^8^ to 2.6 × 10^10^ CFU g^–1^. The solution of polymer and bacteria was used as the dispersed phase, and the emulsification process was carried out as previously described.

### Preparation of Calcium Alginate Microspheres by External Gelation

For the external gelation, two different strategies were evaluated (Figure 2).

**Figure 2:**
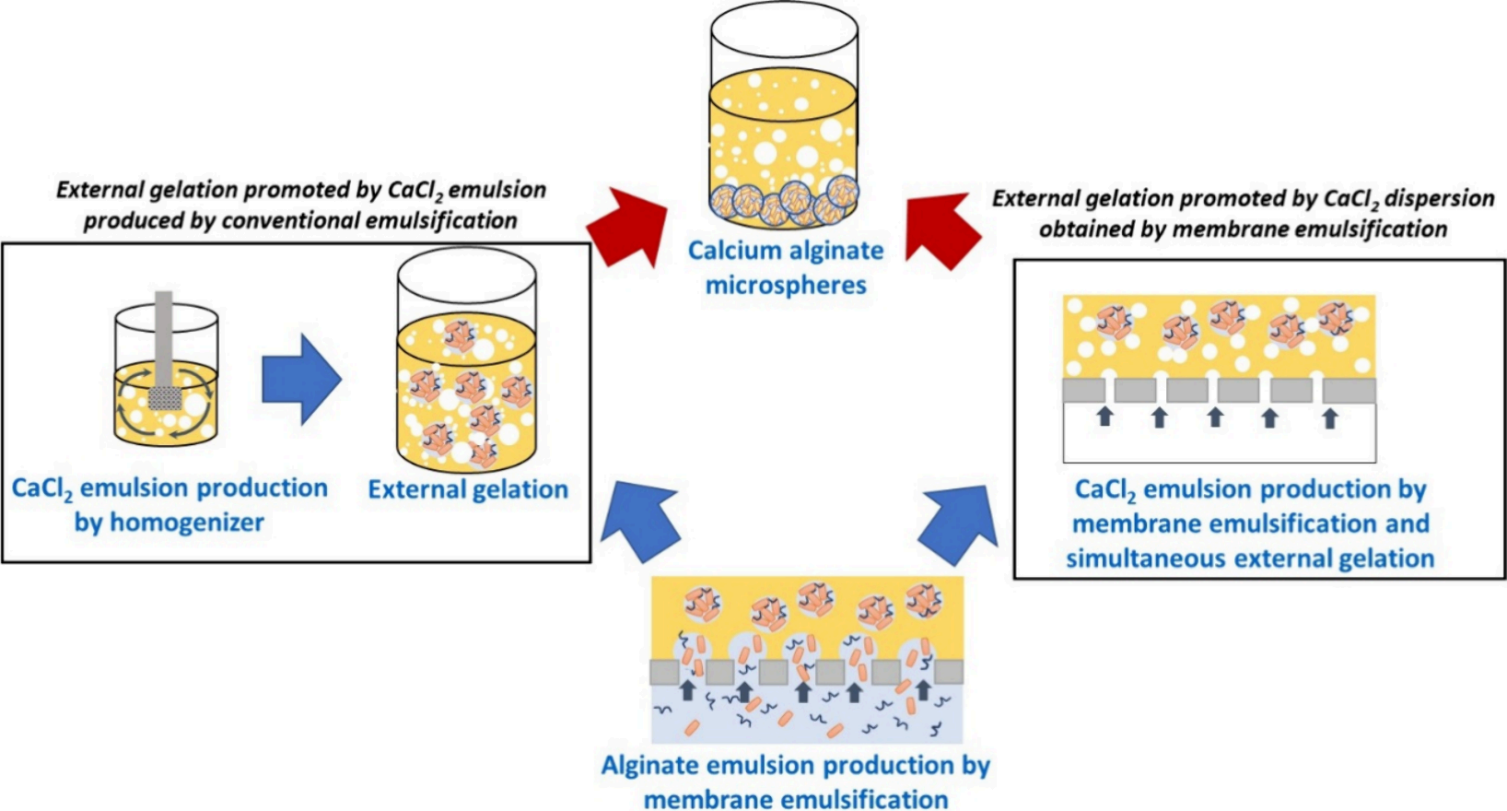
Preparation of calcium alginate microspheres from the alginate emulsion by external gelation promoted by a CaCl_2_ emulsion produced by high-shear conventional emulsification or by the dispersion of CaCl_2_ solution by using membrane emulsification.

In one case, a W/O emulsion was prepared by using CaCl_2_ solution as the dispersed phase and limonene with Span 80 (30 mL) as the continuous phase by using the conventional high-shear emulsification method. The composition and operative conditions used to obtain the cross-linking agent emulsion are summarized in Table 1. The volume and concentration of the CaCl_2_ solution emulsified were in the range from 3 to 15 mL and from 0.5 to 2% w/w, respectively. This emulsion was obtained by the homogenizer (VELP Scientific VO5) by using 10,000–15,000 rpm, in a range of time from 0.5 to 1 min according to the final composition of the CaCl_2_ emulsion. Also, the Span 80 concentration was varied in the range from 0.05 to 2% w/w as a function of the volume of CaCl_2_. The aim was to evaluate whether a different amount of CaCl_2_ solution could influence the cross-linking reaction. The CaCl_2_ emulsion was added drop by drop, under stirring (300 rpm), to the SA emulsion produced by ME. The effect of the time of cross-linking reaction was also evaluated by collecting a sample after 1, 3, 4, and 24 h.

**Table 1:** Effect of CaCl_2_ Emulsion Size and Compositions on Alginate Microspheres

CaCl_2_		CaCl_2_/SA	Span 80	homogenization		CaCl_2_ emulsion		alginate micropheres	
% w/w	mL	mol mol^–1^	% w/w	rpm	min	size (μm)	CV	size (μm)	CV
0.5	3	1	0.05	10,000	0.5	6.6 ± 0.5	0.39 ± 0.03	30 ± 2.1	0.17 ± 0.02
2	1	1	0.05	10,000	0.5	5.2 ± 0.4	0.43 ± 0.03	22.6 ± 1.6	0.13 ± 0.01
2	3	3	0.05	10,000	0.5	5.3 ± 0.4	0.46 ± 0.03	22.8 ± 1.6	0.10 ± 0.01
2	9	9	0.2	10,000	1	4.5 ± 0.3	0.50 ± 0.04	23.8 ± 1.7	0.11 ± 0.02
2	15	15	2	15,000	1	6.9 ± 0.5	0.47 ± 0.03	22.4 ± 1.6	0.13 ± 0.01

An alternative strategy was developed by using the ME to obtain CaCl_2_ dispersion into the SA emulsion previously produced and here used as the continuous phase. A hydrophobic membrane with a pore size of 5 μm was used for this aim in order to promote the fine distribution of CaCl_2_ as a form of uniform droplets directly into the emulsion, where they can contact the alginate droplets. A CaCl_2_ solution with a concentration of 2% w/w was used, and the effect of different volumes (1 and 3 mL) was considered. A dispersed phase flux and shear stress of 7 L h^–1^ m^–2^ and 6.4 Pa were used, respectively. When the desired CaCl_2_/SA emulsion (v/v) was achieved, the process was stopped, and the cross-linking reaction was continued in a separate tank under stirring (300 rpm).

After the cross-linking step, the particles are separated from the solution by centrifugation (6000 rpm, 10 min) and washed with water or Ringer’s solution to rinse off any residual cross-linking agent. According to the composition of the CaCl_2_ emulsion used for the cross-linking, many washing steps were required (2–6 times), followed by the same number of centrifugation cycles.

Particles were observed by using an optical microscope (Motic BA310, Motic Europe, Barcelona, Spain) equipped with a camera (Moticam 10, Motic Europe, Barcelona, Spain), and the diameter was measured by using the Motic Image Plus 3.0 ML software. Data were reported in terms of mean droplet size and coefficient of variation as a result of the measurement of more than 100 droplets for each sample.

The experiments were carried out at least in triplicate, and the results reported are the mean ± standard deviation.

### Encapsulation Efficiency and Microorganism Survival

The encapsulation efficiency (EE) of *K. pseudosacchari* TL13 by ME, followed by external gelation, was evaluated on the emulsions and capsules obtained by the optimized procedure. *K. pseudosacchari* TL13 is a Gram-negative, nonmotile, rod-shaped endophyte (approximately 1 μm wide, 2 μm long).^26^The alginate emulsion was centrifuged (6000 rpm, 25 °C, 10 min) to induce phase separation, while the capsules collected after centrifugation were suspended in Ringer’s solution (10 mL) and homogenized by vortexing for 1 min until they were completely opened. 10-Fold serial dilutions (1:10) were then performed and used to inoculate brain heart infusion (BHI; Oxoid, Milan, Italy) agar plates. After incubation at 30 °C for 24 h, bacterial growth was assessed by determining the number of colonies forming units (CFU). This test was performed for each sample in triplicate. The EE was calculated as the ratio of the number of entrapped bacteria (after emulsification or cross-linking) to the free viable bacteria cells (in the initial alginate solution).

### Bioreactor Fermentation

*K. pseudosacchari* TL13 was also grown in a bioreactor (Applikon MiniBio bioreactors, Getinge, Gothenburg, Sweden) in batch mode with a working volume of 350 mL at 30 °C, pH 6.5 (controlled by the addition of 3 M NaOH and H_2_SO_4_ 15% (v/v)) and 600 rpm. Fermentations were conducted on a simple renewable medium obtained by hydrolyzing orange peel waste. Hydrolysis was conducted inside the bioreactor vessels by resuspending the peels’ powder in 5 mM sodium acetate buffer at a 10% (w/v) biomass load and by maintaining the pH at 5.2 and the temperature at 50 °C; 1% (v/v) of Cellic-CTec2 commercial mix (Novozymes A/S Bagsværd, Denmark) was added for the reaction. After 24 h, the supernatant was recovered by centrifugation at 5000 rpm for 20 min and used as fermentation medium. The medium was supplemented with 13 g L^–1^ of KH_2_PO_4_ to facilitate pH control. During the process, a constant 20% air saturation was maintained by adding pure sterile oxygen to the bioreactor. The broth obtained at the end of the fermentation process was directly frozen at −80 °C for 2 h and freeze-dried by sublimation at −90 °C at a chamber pressure of 1 mbar for 16 h, followed by a secondary drying for 2 h at 0.01 mbar using a bench scale freeze-dryer (Beta 2-8 LSC plus, Christ, Osterode am Harz, Germany). The lyophilized product was dispersed into the alginate solution and used for the microencapsulation experiment as previously described. An initial concentration of 2 × 10^9^ CFU g^–1^ of viable cells in alginate solution was used.

## Results and Discussion

### W/O Emulsion Preparation by ME

The operative conditions of the ME process were optimized before encapsulating *K. pseudosacchari* TL13 in calcium alginate microspheres in order to obtain uniform droplets. The use of limonene as a continuous phase was considered, and the results were compared with those of isooctane. Particles in the same range of size and size uniformity were obtained by using the two solvents in the same conditions of dispersed phase flux and shear stress (Figure 4A), which indicated that limonene can be used to replace isooctane as a renewable solvent in emulsion production. The effect of dispersed phase flux (Figure 4A), shear stress (Figure 3B), and W/O % v/v (Figure 3D) on mean diameter and emulsion uniformity of alginate emulsion droplets was investigated. The mean droplet diameter increased from 50 to 104 μm and decreased from 62 to 43 μm in the range of dispersed phase flux (7–42 L h^–1^ m^–2^) and of shear stress (3.5–10.7 Pa) studied, respectively. These results agreed with previous studies.^27–29^ Uniform droplets (CV lower than 0.2) were produced at the flux and shear stress conditions used. Only when the shear stress was 3.5 Pa was the CV higher than 0.2. Data indicated that the dispersed phase flux can be increased without compromising the uniformity of the emulsion distribution, allowing for an increase in the productivity of the process (intended as the mass of alginate particles obtained) from 140 to 840 g h^–1^ m^–2^. Moreover, the W/O % v/v was also increased from 10 to 30, maintaining the mean diameter and CV quite constant and increasing the alginate concentration in the emulsion from 1.8 to 4.6 g L^–1^.

**Figure 3:**
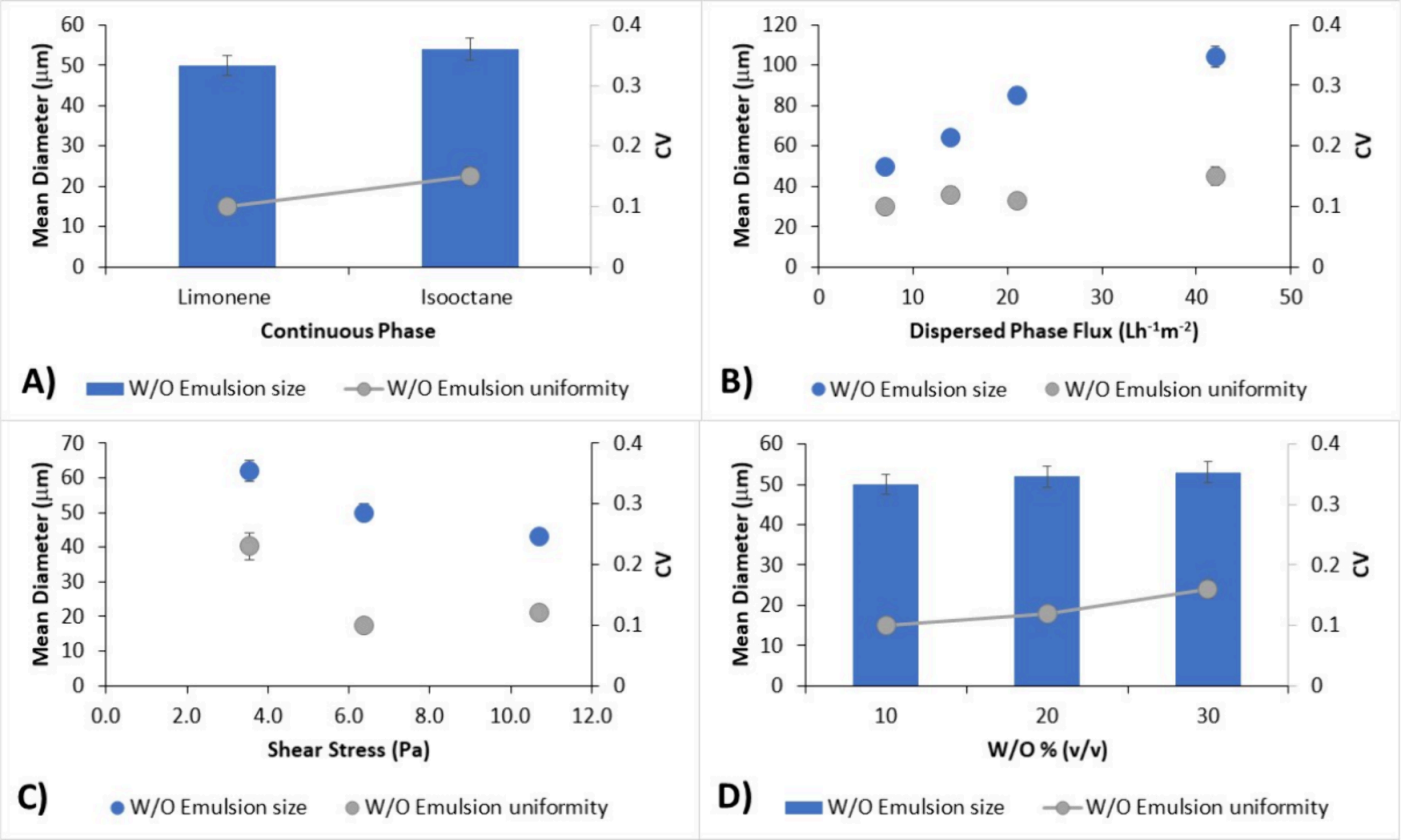
Effect of (A) continuous phase composition (shear stress = 6.4 Pa; dispersed phase flux = 7 L h^–1^ m^–2^; W/O = 10% v/v); (B) dispersed phase flux (shear stress = 6.4 Pa; W/O = 10% v/v), (C) shear stress (dispersed phase flux = 7 L h^–1^ m^–2^; W/O = 10% v/v), and (D) W/O % v/v (dispersed phase flux = 7 L h^–1^ m^–2^; shear stress = 6.4 Pa) on the mean diameter and CV of alginate emulsion.

A dispersed phase flux of 7 L h^–1^ m^–2^ and a shear stress of 6.4 Pa, which allowed the production of uniform droplets with an average diameter of 50 μm and a CV of 0.1, will be used for the preparation of alginate microspheres containing the bacteria.

### Preparation of Calcium Alginate Microspheres by External Gelation

The alginate emulsion was cross-linked with a CaCl_2_ emulsion obtained by homogenizing a certain volume of CaCl_2_ in a Span 80 solution in limonene, as specified in Table 1. In this process, Ca^2+^ ions were in contact with the external surface of alginate droplets in the form of small droplets, increasing their surface area and promoting Ca^2+^ diffusion through the continuous phase.^30^

The effect of cross-linking reaction time was evaluated (Figure 4). A slight decrease in the alginate microsphere size as a function of time was observed during 4 h of reaction. The size of capsules decreased from 26 to 22.8 μm, which corresponds to a particle shrinkage from 48 to 55% in respect to the emulsion droplet size. This behavior can be correlated with a typical mechanism of external gelation. Upon contact between the two emulsions, the cross-linking occurred at the periphery of the alginate droplet, while their extended contact allowed for further diffusion of Ca^2+^ across the external gelled layer via a concentration gradient, subsequently leading to the solidification of the droplet core.^25^ A 4 h time was chosen for the cross-linking step based on optimization experiments.

**Figure 4:**
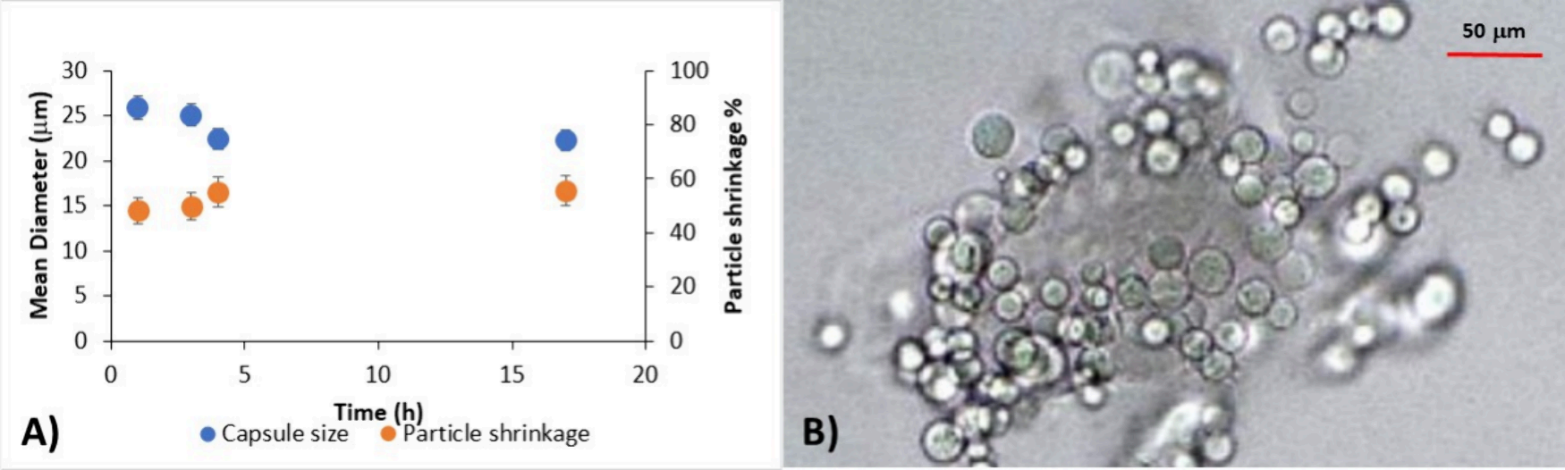
(A) Effect of cross-linking reaction time on mean diameter and particle shrinkage of alginate microspheres. (B) Microphotograph of alginate microspheres obtained by ME and external gelation (CaCl_2_/SA molar ratio = 3; cross-linking reaction time = 4 h).

The composition of the Ca^2+^ emulsion was modified to evaluate the influence on the cross-linking process (Table 1). Independent of the concentration and volume of the CaCl_2_ solution emulsified, CaCl_2_ droplets’ size was almost in the same range (5.7 ± 0.9 μm), which was around 9 times the size of the SA droplets (50 μm). Alginate microspheres with a size of about 22.8 ± 0.6 μm and spherical shape (Figure 4B) were obtained, which were properly hardened, as demonstrated by their resistance to deformation during recovery and washing procedures applied after the cross-linking process. When the CaCl_2_/SA molar ratio was decreased to 1 (by using a CaCl_2_ concentration of 0.5% w/w and a volume of 3 mL), a certain increase in the alginate microspheres' size was observed (up to 30 μm) as a result of low cross-linking between the alginate polymer chains and Ca^2+^ ions. It is possible that a low CaCl_2_/SA molar ratio determined the formation of a semisolid membrane at the periphery of the alginate droplet with a more liquid core,^31^ while the immersion of alginate droplets in a more concentrated cross-linking agent allowed for further diffusion of Ca^2+^ across the membrane, leading to the extension of the solidification to the droplet core. Moreover, data indicated that increasing the CaCl_2_/SA molar ratio up to 3 is not required, as properly structured microspheres were obtained by using a CaCl_2_ solution with a concentration of 2% w/w and a volume of 3 mL. Similar results were also achieved by decreasing the CaCl_2_ solution volume to 1 mL and maintaining a concentration of 2% w/w, although a low CaCl_2_/SA molar ratio was used (equal to 1). This indicated that the reaction between the alginate droplet and CaCl_2_ solution at the droplet–droplet interface can be optimized by using an appropriate concentration of cross-linking agent that directly interacts with polymer chains.

Recovery of alginate microspheres from the oil environment after internal and external gelation is an important step of the process.^32^ Both separation time and volume of the aqueous phase may limit the development of the process at large scale.^32^ The decrease of Span 80 concentration (to 0.05% w/w) and of CaCl_2_ solution volume (1 and 3 mL) allowed shortening of the separation of the microspheres from the cross-linking reaction environment, simplifying the overall production procedure.

Results indicated that ME in combination with external gelation by using CaCl_2_ emulsion resulted in a promising method for large-scale production of alginate gel beads. To simplify the scale-up and make the process continuous, the use of ME was also evaluated to directly disperse the CaCl_2_ solution into the SA emulsion. CaCl_2_ solution with a concentration of 2% w/w was used, and the effect of different volumes (1 and 3 mL) was considered (Table 2).

**Table 2:** Effect of CaCl_2_ Dispersion SA Emulsion by Membrane Emulsification on Alginate Microspheres

CaCl_2_		CaCl_2_/SA	Span 80	membrane pore size	CaCl_2_ emulsion		alginate micropheres	
% w/w	mL	mol mol^–1^	% w/w	μm	size (μm)	CV	size (μm)	CV
2	1	1	0.05	5	12.2 ± 0.9	0.15 ± 0.01	22.3 ± 1.6	0.15 ± 0.01
2	3	3	0.05	5	12.6 ± 0.9	0.17 ± 0.01	22.5 ± 1.4	0.17 ± 0.01

The use of the ME process to disperse the CaCl_2_ solution into the SA emulsion permitted the production of alginate microspheres in the same range of size and with comparable uniformity to the homogenizer, simplifying the procedure and achieving a good control of the cross-linking reaction in the biphasic system. CaCl_2_ droplets, produced drop-by-drop by the membrane with a 5 μm pore size, were small enough to create a larger interface area where the migration of CaCl_2_ to the alginate emulsion interface occurred. Moreover, the use of two ME units connected in series allows drop generation and external gelation reaction in a continuous mode.

### Encapsulation Efficiency and Microorganism Survival

The success of using PGPM introduced into the soil requires the survival of an adequate number of bacteria. For this reason, in the present work, the encapsulation efficiency of *K. pseudosacchari* TL13 was evaluated as a function of the initial value of the bacterial concentration and both after emulsification and cross-linking reaction carried out in the optimized conditions, which are key factors in the success of microbial encapsulation.

Previous works on PGPM encapsulation do not specify the concentration of microorganisms encapsulated, and when this value is reported, there is a large discrepancy in the data (typical values are in the range from 10^6^ to 10^12^ CFU g^–1^).^1^ PGPM encapsulation yield is extremely variable, and the relatively low CFU counts of the capsuled-entrapped biomass often reported can be correlated with the losses in bioactivity during the encapsulation process (for alginate beads gelification and hardening times).^33^

Various concentrations of *K. pseudosacchari* TL13 were mixed with alginate solution in order to have initial concentrations of viable cells of 4.4 × 10^8^, 4.4 × 10^9^, and 2.6 × 10^10^ CFU g^–1^ (Figure 5). The experiments were carried out keeping both alginate concentration and operative conditions used for emulsification and cross-linking constant. The influence of the membrane-based emulsification method was specifically studied to evaluate the effect of bacterial translocation across membrane pores on strain viability.

**Figure 5:**
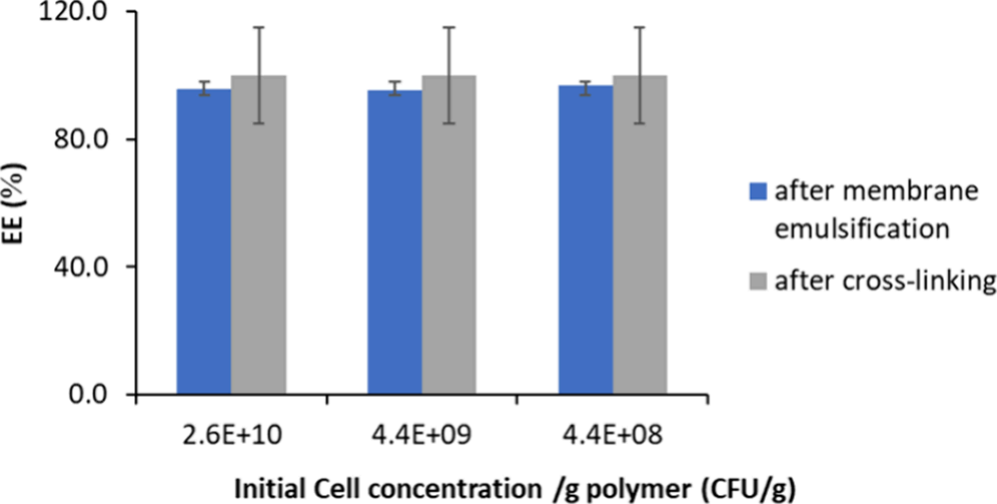
Encapsulation efficiency after emulsification and cross-linking as a function of initial viable cell concentration.

The EE ranged from 95.5 ± 2.5 to 97.0 ± 2.8% for all the initial microbial concentrations, indicating that ME was a suitable method to preserve microorganisms' vitality and achieve high encapsulation efficiency. This was in line with previous works in which encapsulation of living cells by ME was studied.^8,9,11^ However, Camelo-Silva et al. reported a final EE ≥ 93% referred to the capsules obtained after solidification of liquid droplets^8^ without specifying the EE at the end of the emulsification step. Only Morelli et al. specifically demonstrated that yeast cells were not retained by nontortuous pore channels, giving an encapsulation efficiency of 100%.^9^ Overall, data indicated that ME is a promising encapsulation method for the encapsulation of living cells: the fluid dynamic conditions used during the process do not compromise bacterial survival, and the drop-by-drop mechanism guarantees the encapsulation effectiveness. The EE achieved after the cross-linking step was also evaluated, and a significant variation was observed, indicating that the emulsification step is the limiting step, causing a decrease in cell viability as a result of the low shear stress applied (Figure 5).

### Fermentation Process Efficiency

It is by now clear that the use of microbial biostimulants as tools for modern and sustainable agricultural practices is crucial; however, several challenges for a broad and established application exist.^34^ Among them is the efficiency and economic viability of the manufacturing process that involves various steps from medium design to biomass preservation during conservation and formulation for delivery. Considerations on the first step include using low-cost fermentation substrates that maximize biomass production and process efficiency in terms of biomass conversion to fermentable sugars and yields of microbial biomass obtained. The latter were considered to evaluate the overall effectiveness of the process applied in this study.

The percentage of cellulose and hemicellulose in orange peels is reported to vary between 9 and 37 and 10–18%, respectively.^35–42^ Considering an average of the highest values found in the literature, namely 34.13 ± 2.9% for cellulose and 12.9 ± 4.1%, the enzymatic digestion of the waste biomass provided from a juice manufacturing company (Gioia Succhi, Calabria, Italy) converted approximately 88% of cellulose and 79% of hemicellulose into glucose and xylose, respectively, according to eqs 1 and 2.

$$\mathrm{cellulose}\;\mathrm{conversion}\;(\%)=\frac{\left[\mathrm{glucose}\right]}{[\mathrm{biomass}]\times F_{\mathrm{cell}}\times 1.11}\times 100$$ 1

$$\mathrm{hemicellulose}\;\mathrm{conversion}\;(\%)=\frac{\left[\mathrm{hylose}\right]}{[\mathrm{biomass}]\times F_{\mathrm{hcell}}\times 1.14}\times 100$$ 2

The glucose consumed during the process was converted to 8.0 ± 0.4 g L^–1^ of dry biomass and 9.4 ± 1.4 g L^–1^ of exopolysaccharide (EPS), corresponding to a *Y*_X/S_ of 0.25 g g^–1^ and a *Y*_P/S_ of 0.30 ± 0.04 g g^–1^. The medium and fermentation conditions used addressed metabolism concurrently toward two added-value products, namely, biomass and EPS, to exploit both during processing and combine them in the final formulation. *Kosakonia* strains commonly produce fucose-rich heteropolysaccharides (containing also glucose, galactose, glucuronic acid, and pyruvic acid) with several potential applications in the biotechnological field.

Considering biomass, EPS, acetic acid, and lactic acid as main fermentation products, and glucose as the only carbon source consumed (xylose was not consumed), the carbon balance closed at 0.78 C-mol C-mol^–1^ glucose (Table 3), indicating that about 22% of the carbon was present in unmeasured carbon-containing products; considering that *K. pseudosacchari* uses an aerobic metabolism, probably most of this carbon was released as CO_2_, which normally represents a substantial portion of carbon efflux in glucose metabolism, thereby closing the balance.

**Table 3:** Carbon Balance of the Fermentation Processes Performed to Obtain the *K. pseudosacchari* TL13 Biomass Used for Encapsulation Experiments

glucose consumed (g L^–1^)	C fraction of substrates consumed (g)	C fraction of biomass^a^ produced (g)	C fraction of EPS^b^ produced (g)	C fraction of LA produced (g)	C fraction of AA produced (g)	*Y*_C_ = X/S (g g^–1^)	*Y*_C_ = LA/S (g g^–1^)	*Y*_C_ = EPS/S (g g^–1^)	*Y*_C_ = AA/S (g g^–1^)	carbon balance outlet/inlet
31.4 ± 0.14	13.08 ± 0.06	3.9 ± 0.3	3.8 ± 0.13	1.1 ± 0.4	1.5 ± 0.3	0.30	0.13	0.29	0.11	0.78

a Biomass elemental composition CH_1.8_O_0.5_N_0.2_ based on Neidhardt et al.^43^

b EPS composition (data not shown). *Y*_C_ = X/S, carbon yield of biomass on glucose, considering carbon moles present in substrate and product; *Y*_C_ = EPS/S, carbon yield of EPS on glucose, considering carbon moles present in substrate and product; *Y*_C_ = AA/S, carbon yield of acetic acid on glucose, considering carbon moles present in substrate and product. *Y*_C_ = LA/S carbon yield of lactic acid on glucose, considering carbon moles present in substrate and product.

The global fermentation process efficiency (η_global_), considering the conversion efficiency of cellulose into glucose (η_cellglu_ = 0.88 g g^–1^) and the conversion efficiency of glucose into biomass and EPS (η_gluX_EPS_ = 0.25 + 0.30 = 0.55 g g^–1^), was equal to 0.48 g g^–1^. This result indicates that about half of the cellulose is converted into added-value products. *Kosakonia cowanii* grown on sugar cane juice supplemented with yeast extract and NaNO_3_ showed a high *Y*_P/S_ of 0.52 g g^–1^ and, on the other hand, a low *Y*_X/S_ of 0.06 g g^–1^.^43,44^ In the present study, the combination of high hydrolysis efficiency and high total added-value product yields makes this process an efficient and sustainable microbial route for converting cellulose into biostimulant biomass enriched with EPS. In fact, considering its antimicrobial potential and contribution to microbial attachment to surfaces,^45^ the EPS might also be useful to help the microbial biostimulant in the first stages of rhizosphere colonization.

### Sustainable and Integrated Bioreactor Fermentation and Membrane Emulsification for the Production of *K. pseudosacchari* TL13-Based Biostimulant

Different types of factors affect the success of the application of microbial biostimulants. Here, we focused on two aspects regarding overall process sustainability and bioformulation efficiency. The first aspect was addressed by finding green solutions throughout the development process: (1) the upstream process was based on the use of renewable waste that does not require biomass pretreatments, often generating pollutants (e.g., concentrated acids/bases) and that is efficiently hydrolyzed, resulting in high yields of fermentable sugars. The medium for fermentation was entirely based on the hydrolysate; no additional nitrogen source was supplemented, strongly reducing costs and addressing metabolism to the production of high concentrations of biomass and EPS, both added-value products; (2) the downstream process was simplified by avoiding separation steps at the end of the fermentation process, thereby using the whole broth as material for biostimulant production. This produces several advantages; in fact, besides lowering downstream and waste disposal costs, the presence of the EPS is functional to protect cells, not only during the following processing, but also during storage of the final product. High EPS concentrations in the broth protected cells during lyophilization as efficiently as trehalose (data not shown), acting as stabilizers and avoiding the addition of adjuvants normally added to the formulations to enhance strain survival.^46^ Moreover, as previously mentioned, the presence of the EPS in the formulation may also favor plant microbe interactions and improve microbial resistance to environmental conditions affecting strain's ability to adhere to surfaces and to compete with other microorganisms.

The bioreactor fermentation was integrated with the membrane emulsification process to produce alginate capsules containing the lyophilized product of the fermentation, as previously described (Figure 6). The optimized conditions permitted to obtain particles in the same range of size (25 ± 1.3 μm) and with an encapsulation efficiency of 94.9 ± 10.6% starting from an initial concentration of 2 × 10^9^ CFU g^–1^ of viable cells in alginate solution. Due to the high reproducibility of the ME process,^16^ this result was expected but not obvious, considering that usually the payloads are pure substances or cells cultured in a defined medium. To the best of our knowledge, this is the first case in which the ME process was used to encapsulate microbial biomass and the whole broth (including EPS) without additional downstream steps. Previously, the polyphenolic compounds purified and concentrated from a real stream (the olive mill wastewaters) were successfully encapsulated in a water-in-oil emulsion by ME.^47^

**Figure 6:**
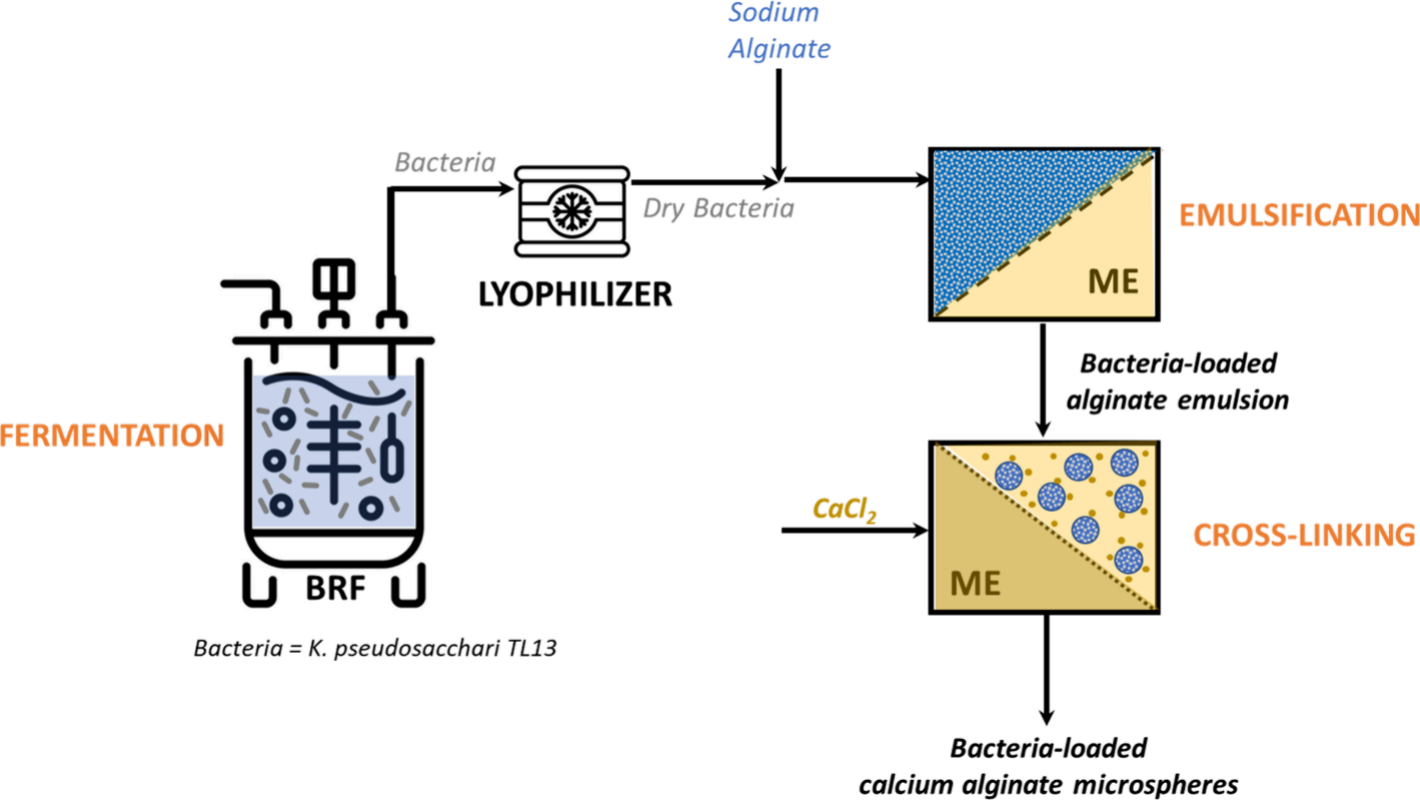
Integrated bioreactor fermentation (BRF) and membrane emulsification (ME) for the production of the *K. pseudosacchari* TL13-based biostimulant.

A measure of the environmental impact of the upstream process for bioreactor fermentation indicates an environmental-factor (*E*-factor) of 6.2 if the orange peel waste is considered among the total mass of waste generated according to eq 3:

E‐factor=[wastemass(orangepeel+Enz+Buf+salt)−products(microbialbiomass+EPS)]/[products(microbialbiomass+EPS)] 3

Considering the lignocellulosic material as a pre-existing waste (eq 4), the *E*-factor is equal to 0.46.

E‐factor=[wastemass(Enz+Buf+salt)−products(microbialbiomass+EPS)]/[products(microbialbiomass+EPS)] 4

The evaluation of the environmental impact of biological processes can be quite complex, and only a few references are available. A thorough calculation of the complete *E*-factor (equal to 37.835) for enzyme production and purification from *Escherichia coli* BL21 was reported by Becker and colleagues.^48^ A simpler calculation was performed here; however, clearly, the simplicity of the fermentation medium and the lack of a purification process strongly reduce the *E*-value.

The E-factor was also calculated for the membrane emulsification process and cross-linking reaction optimized to produce the encapsulated *K. pseudosacchari* TL13 according to eq 5.^49–51^

E‐factor=[rawmaterials(polymer+microbialbiomass+EPS)+reagent(emulsifier+crosslinkingagent)−products(capsulescontainingmicrobialbiomass+EPS)]/[products(capsulescontainingmicrobialbiomass+EPS)] 5

For particle production, it is also relevant to consider the contribution of the solvent (including water) and the complete *E*-factor as well as % solvent + water (in respect to the total mass of reagents).^50,51^ Results are summarized in Table 4, and the data used for calculations are reported in the Supporting Information.

**Table 4:** Green Factors Related to the Production of *K. pseudosacchari* TL13-Loaded Alginate Microspheres by Membrane Emulsification and CaCl_2_ External Gelation

component	total mass (mg)	sEF	cEF	% cEF litd	% solvent + water
raw materials (sodium alginate, *K. pseudosacchari* TL13)	6.00 × 10^1^	10.6	950.6	1.11	98.78
reagents (Span 80, CaCl_2_)	6.35 × 10^1^				
solvent (limonene)	5.05 × 10^4^				
water	5.94 × 10^3^				

An *E*-factor (sEF) and a complete *E*-factor (cEF) equal to 10.6 and 950 were achieved, respectively, which are in line with previous data calculated for the production of polymeric particles by membrane emulsification.^50,51^ % solvent + water results in 98.78, which indicates the high contribution of these components to the production of alginate microspheres. Considering that limonene is a greener solvent with respect to the petroleum-derived solvents conventionally used for emulsification processes, it is possible to assume that overall, the microspheres productive process is sustainable. Moreover, if we consider the biostimulants as a part of the fine chemical industry and that the *E*-factor in this segment of the chemicals industry typically ranges from 5 to 50,^52^ the *E*-factor of the integrated bioreactor fermentation and membrane emulsification process indicates that their integration can promote pollution prevention and sustainable biostimulant production.

The proposed integrated process demonstrated industrialization potential that could be further enhanced by introducing an appropriate strategy to purify and separate the microspheres after their production. In the present work, multiple centrifugation and rinsing steps are used for the practical aim of characterizing the particles; however, this approach is unlikely to be a viable route for large-scale production. An effective alternative could be based on the use of standard freeze-drying procedures or membrane filtration.

## Conclusions

Despite biostimulants' sustainability benefits, they still face barriers to widespread agricultural use and require further innovation in formulation and production. In the present work, the potential of an integrated production process combining bioreactor fermentation with the microencapsulation (ME) process to produce and encapsulate *K. pseudosacchari* TL13 as a promising biofertilizer was evaluated.

ME is proposed as an innovative and sustainable method for producing alginate microspheres for encapsulation of *K. pseudosacchari* TL13. The data indicated that (i) limonene can be used to replace isooctane as a renewable solvent in emulsion production, (ii) the mean droplet diameter increased from 50 to 104 μm and decreased from 62 to 43 μm in the range of dispersed phase flux (7–42 L h^–1^ m^–2^) and of shear stress (3.5–10.7 Pa) studied, (iii) the dispersed phase flux can be increased without compromising the uniformity of the emulsion distribution allowing to increase the productivity of the process, (iv) the W/O % v/v was also increased from 10 to 30 while maintaining the mean diameter and CV quite constant increasing the alginate concentration in the emulsion from 1.8 to 4.6 g L^–1^. The use of two ME units, one for the production of SA emulsion and the other for CaCl_2_ emulsion, connected in series, permitted the achievement of drop generation and external gelation reaction in a continuous mode, which seems to be a promising method for large-scale production of alginate gel beads. A high encapsulation efficiency (EE > 95%) was obtained.

The bioreactor fermentation, using orange peel waste as a substrate for microorganism fermentation, converted more than half of the cellulose into biomass and added-value products. The combination of high hydrolysis efficiency and high total added-value product yields makes this process an efficient and sustainable microbial route for converting cellulose into biostimulant biomass enriched with EPS that was encapsulated by ME.

The developed integrated process could promote the widespread adoption of *K. pseudosacchari* TL13 or other PGPM as a sustainable and efficient method for microbial biostimulants production and formulation.

## Supplementary Material



## Notes

Definitions of the parameters used to evaluate the membrane emulsification greenness and data used for calculations (PDF)
