# Supplementary material for: Sustainable Microbial Biostimulant Production by Integrated Bioreactor Fermentation and Membrane Emulsification
Source: ACS Sustain Chem Eng. 2026 May 5;14(19):9306–16. doi: 10.1021/acssuschemeng.6c02820 (PMC13195560; doi:10.1021/acssuschemeng.6c02820)
Supplement: Supplementary file 1 [file sc6c02820_si_001.pdf]

Supplementary Material

Sustainable Microbial Biostimulant Production by integrated Bioreactor Fermentation and Membrane Emulsification

Emma Piacentini[a*], Antonio Condello[a], Fabio Bazzarelli[a], Rebecca Italia[a], Valeria Ventorino[b], Donatella Cimini[c],

[a]Institute on Membrane Technology (CNR-ITM), Rende (CS), Italy

[b]Department of Agricultural Sciences, University of Naples Federico II, Portici, Italy

[c]Department of Environmental, Biological and Pharmaceutical Sciences and Technologies, University of Campania L. Vanvitelli, Caserta, Italy

[*]Corresponding Author

Emma Piacentini: e.piacentini@itm.cnr.it

Green factor calculation

The greenness of the process was determined via complete E factor calculation based on a simple mass-balance as previously reported. Data used for the estimation are summarized in table S1.

The equations used to calculate Simple E factor (sEF), Complete E factor (cEF), %cEF and %solvent+water are reported in the following:

$$\mathrm{sEF} = \frac{\sum \mathrm{m(Raw\ Materials)} + \sum \mathrm{m(Reagents)} - \mathrm{m(Product)}}{\mathrm{m(Product)}}$$

$$cEF = \frac{\sum m(Raw\ Materials) + \sum m(Reagents) + \sum m(Solvents) + \sum(Water) - m(Product)}{m(Product)}$$

$$\%cEF = \frac{sEF}{cEF} * 100\%$$

$$\%solvent + water = \frac{m(solvent) + m(water)}{m(total)}$$

Table S1. Data used for greenness evaluation.

Classification	Material	Volume (mL)	Mass (mg)	Concentration or Density (mg/mL)
**Raw Materials**	Alginate	3	60	20
	Kosaconia	3	0.0003	0.0001
**Reagent**	Span 80	30	600	20
		30	15	0.5
	CaCl2	1.00	20	20
**Solvent**	limonene	30.00	25230	841
	limonene	30.00	25230	841
**Water**	Water	6.00	5940	1000

Green Analysis

**m(Raw Materials) mg**	6.00E+01
**m(Reagents) mg**	6.35E+02
**m(Solvents) mg**	5.05E+04
**m(Water) mg**	5.94E+03
**Total**	5.71E+04
**m(Product) mg**	6.00E+01

**sEF**	10.6
**cEF**	950.6
**% cEF litd**	1.11%
**% solvent + water**	98.78%
